# Activation of multiple receptors stimulates extracellular vesicle release from trophoblast cells

**DOI:** 10.14814/phy2.14592

**Published:** 2020-10-20

**Authors:** Kirk P. Conrad, Kubra M. Tuna, Cathleen T. Mestre, Esha S. Banwatt, Abdel A. Alli

**Affiliations:** ^1^ Department of Physiology and Functional Genomics University of Florida College of Medicine Gainesville FL USA; ^2^ Department of Obstetrics and Gynecology University of Florida College of Medicine Gainesville FL USA; ^3^ D. H. Barron Reproductive and Perinatal Biology Research Program University of Florida Gainesville FL USA; ^4^ Department of Medicine, Division of Nephrology, Hypertension and Renal Transplantation University of Florida College of Medicine Gainesville FL USA

**Keywords:** angiotensin, bitter taste receptor, calcium, cholecystokinin, exosome, GPCR, muscarinic receptor, placenta

## Abstract

Reports of the stimulated release of extracellular vesicles (EVs) are few, and the mechanisms incompletely understood. To our knowledge, the possibility that the activation of any one of the multitudes of G‐protein‐coupled receptors (GPCRs) expressed by a single cell‐type might increase EV release has not been explored. Recently, we identified the expression of cholecystokinin (CCK), gastrin, gastrin/cholecystokinin types A and/or B receptors (CCKAR and/or –BR), and the bitter taste receptor, TAS2R14 in the human and mouse placenta. specifically, trophoblast. These GPCR(s) were also expressed in four different human trophoblast cell lines. The current objective was to employ two of these cell lines—JAR choriocarcinoma cells and HTR‐8/SVneo cells derived from first‐trimester human villous trophoblast—to investigate whether CCK, TAS2R14 agonists, and other GPCR ligands would each augment EV release. EVs were isolated from the cell‐culture medium by filtration and ultracentrifugation. The preparations were enriched in small EVs (<200 nm) as determined by syntenin western blot before and after sucrose gradient purification, phycoerythrin (PE)‐ADAM10 antibody labeling, and electron microscopy. Activation of TAS2R14, CCKBR, cholinergic muscarinic 1 & 3, and angiotensin II receptors, each increased EV release by 4.91‐, 2.79‐, 1.87‐, and 3.11‐fold, respectively (all *p *< .05 versus vehicle controls), without significantly changing EV diameter. A progressive increase of EV concentration in conditioned medium was observed over 24 hr consistent with the release of preformed EVs and de novo biogenesis. Compared to receptor‐mediated stimulation, EV release by the calcium ionophore, A23187, was less robust (1.63‐fold, *p* = .08). Diphenhydramine, a TAS2R14 agonist, enhanced EV release in JAR cells at a concentration 10‐fold below that required to increase intracellular calcium. CCK activation of HTR‐8/SVneo cells, which did not raise intracellular calcium, increased EV release by 2.06‐fold (*p* < .05). Taken together, these results suggested that other signaling pathways may underlie receptor‐stimulated EV release besides, or in addition to, calcium. To our knowledge, the finding that the activation of multiple GPCRs can stimulate EV release from a single cell‐type is unprecedented and engenders a novel thesis that each receptor may orchestrate intercellular communication through the release of EVs containing a subset of unique cargo, thus mobilizing a specific integrated physiological response by a network of neighboring and distant cells.

## Graphical abstract

Activation of multiple G protein‐coupled receptors stimulated extracellular vesicle release from a single cell‐type trophoblast‐derived cells. Other cell signaling pathways may underlie receptor‐stimulated extracellular vesicle release besides, or in addition to, calcium.

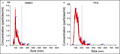

## 1 INTRODUCTION

In previous work, we revealed the expression of cholecystokinin (CCK), gastrin, and their receptors gastrin/cholecystokinin types A and/or B receptors (CCKBR and/or AR) in trophoblast cells of human and mouse placenta by RT‐PCR and immunohistochemistry. The bitter taste receptor, TAS2R14, was also identified in the human placental trophoblast cells. In addition, these hormones and receptors were identified in several choriocarcinoma trophoblast‐derived cell lines, and in HTR‐8/SVneo, an immortalized first trimester placental trophoblast cell line. Both CCKBR and TAS2R14 agonists increased intracellular calcium in these human trophoblast cell‐lines with the notable exception of HTR‐8/SVneo, in which CCK was ineffective in this regard (Taher et al., 2019). The physiological role(s) and potential cross‐talk in normal and pathological placentas of these hormones and receptors are unknown, but by analogy to other organ systems, one possibility is that they may contribute to immunologic regulation at the maternal‐fetal interface [citations in Taher et al., (2019)]. Although not mutually exclusive, another intriguing possibility is that they may regulate extracellular vesicle release, which has been linked to calcium signaling (Faure et al., 2006; Savina, Furlan, Vidal, & Colombo 2003).

Extracellular vesicles (EVs) are characterized according to diameter size as small EVs (<100 nm or <200 nm) and medium/large EVs (>200 nm) (Thery et al., 2018; Witwer & Thery, 2019). Exosomes are bilipid membrane‐bound EVs ranging from 30 to 100 nm in diameter as originally described (Johnstone, Adam, Hammond, Orr, & Turbide, 1987), and in more recent reports, 50–150 nm (Jeppesen et al., 2019; van Niel, D'Angelo, & Raposo, 2018) or up to 200 nm diameter (Yi et al., 2020). Exosomes arise from late‐stage endosomes leading to the formation of multivesicular bodies (MVBs) containing small intraluminal vesicles (ILVs). The MVBs fuse with the plasma membrane releasing the small intraluminal vesicles as EVs called exosomes (<200 nm diameter) into the extracellular space. Other EVs designated microvesicles (>200 nm diameter) derive by budding from the plasma membrane (van Niel, D'Angelo, & Raposo, 2018). [It should be noted that there is likely to be an overlap of diameters among EV populations, that is, the diameters listed above are approximations (Thery et al., 2018; Witwer & Thery, 2019).]

Extracellular vesicles can contain a variety of molecular cargo including proteins, RNA, and lipids. The cargo of exosomes appears to be selectively packaged in the ILVs within the MVB, which can regulate the function of neighboring and distant cells (van Niel, D'Angelo, & Raposo, 2018). Recently, EVs have been linked to many physiological functions and pathological disease states, in which they present promising potential as diagnostic biomarkers and therapeutic vehicles (Shah, Patel, & Freedman, 2018). During pregnancy, elevated numbers of placental‐derived EVs have been found in the peripheral circulation of pregnant women (Sarker et al., 2014). Syncytiotrophoblast has been identified as the primary source of these EVs, which have been proposed to play a role in fetal‐maternal immune tolerance and communication, and possess anti‐viral activity (Delorme‐Axford et al., 2013; Dragovic et al., 2015).

Stimulated extracellular vesicle release is relatively understudied and the mechanisms incompletely understood. There are only a few reports about EV release by the activation of G protein‐ coupled receptors (GPCRs) in different cell types: carbachol stimulation of the muscarinic receptor type 1 expressed in human T cell leukemia Jurkat cells (Alonso et al., 2005); angiotensin II activation of the Type‐1 Angiotensin II Receptor (AT1R) stably expressed in HEK293T cells (Pironti et al., 2015); and histamine stimulation of receptors in HeLa cells (in this case, assessed as the number of MVB‐plasma membrane fusion events) (Verweij et al., 2018). To our knowledge, however, the possibility that the activation of any one of the many GPCRs expressed by a single cell‐type might lead to an increase of EV release has not been previously explored. Because there are as many as 800 GPCRs in the human genome, many of which can be expressed by one given cell‐type (Kroeze, Sheffler, & Roth, 2003), conceivably each GPCR (or perhaps a cohort of functionally related GPCRs) could affect EV release in a cell‐type‐specific manner containing a unique subset of cargo (e.g., specific RNA, protein, and lipid molecules) that, in turn, coordinates specific physiological responses among neighboring and distant cells. Human trophoblast express TAS2R14 and CCKBR (vide supra), as well as cholinergic receptor muscarinic 1 & 3 (CHRM1/3), and type‐1 and −2 angiotensin II receptor (AGTR1 and –R2) (Pavia et al., 1997; Taher et al., 2019; Tower, Lui, Charlesworth, Smith, Aplin, & Jones, 2010). Thus, we employed human trophoblast cell‐lines as a model system to test the hypothesis that the activation of each one of these GPCRs can increase the release of EVs.

## 2 METHODS

### 2.1 Cell culture and treatment

We employed the JAR choriocarcinoma cell line—a human trophoblast‐derived cell, and HTR‐8/SVneo trophoblast cell line derived from first‐trimester human villous trophoblast, because they were previously shown to express four GPCRs—TAS2R14, CCKBR, CHRM1/3, and AGTR1 and –R2 (as described in the Introduction). Thus, these cells were a convenient experimental model in which to test our hypothesis that the activation of many different receptors in one cell type would lead to the release of EVs. JAR cells were initially seeded from vials maintained in liquid nitrogen containing 1 million cells per 1.0 ml of FBS with 10% DMSO as a cryopreservative. They were placed into a T75 flask with 12–15 ml of growth medium—RPMI 1,640 with glucose (2.0 g/L), L‐glutamine (0.3 g/L), sodium bicarbonate (2.0 g/L), penicillin‐streptomycin, 10% FBS—and grown at 37˚C in 5% CO_2_/balance room air with replenishment of growth medium every two days. Upon reaching ~ 90%–100% confluency, cells were split into several T75 and/or T150 flasks, in order to generate 35–45 ml of conditioned medium per treatment for the isolation of EVs (see EV Isolation, below). Seeding density for a T‐75 flask was typically ~ 375,000 cells/flask (5,000 cells/cm^2^).

The HTR‐8/SVneo cells were generously provided by Dr. Charles Graham, Queens University (Graham et al., 1993). We previously corroborated the trophoblast origin of these cells in our laboratory (Ogunleye, Campo, Herrera, Post Uiterweer, &Conrad, 2017). HTR‐8/SVneo cells were cultured in T75 flasks containing complete medium: RPMI 1,640 with glucose (2.0 g/L), L‐glutamine (0.3 g/L), sodium bicarbonate (2.0 g/L), penicillin‐streptomycin, and 10% FBS.

Upon reaching 75%–90% confluency, JAR or HTR‐8/SVneo cells were treated with agonist or vehicle control for ~ 24 hr unless otherwise indicated. Conditioned medium was then collected into 50 cc tubes. Cells were subsequently scraped from flasks using RIPA buffer and protease inhibitors, and the lysates stored at −20 ˚C for BCA Protein Assay.

The receptor ligands, concentrations, and vehicles used in this work are listed in Table 1.

**Table 1:** GPCR receptors and agonists

Agonist	Receptor	Concentration stock/final	Vehicle control
FFA	TAS2R14‐Bitter Taste Receptor	30 mM/30 µM	Dilute DMSO in medium
Chlorhexidine	TAS2R14‐Bitter Taste Receptor	10 mM/10 µM	Medium, only
Diphenhydramine	TAS2R14‐Bitter Taste Receptor	34.3 mM/30 µM	Medium, only
sCCK	Gastrin/Cholecystokinin type B Receptor	0.1 mM/0.1 µM	Vehicle for sCCK in medium
Angiotensin II	Angiotensin II Type−1 Receptor	1.0 mM/0.1 µM	Medium, only
Xanomeline	Cholinergic Muscarinic Receptor Subtypes 1/3	2.7 mM/1.0 nM & 3.0 nM	Dilute DMSO in medium

Note

FFA, flufenamic acid; sCCK, sulfated cholecystokinin. DMSO, diluted to the same degree as stock FFA to make the final concentration. Vehicle for sCCK: 1:1:40 methanol:0.1 M acetic acid: water. Cultured cells were treated for 24 hr with various GPCR agonists or their respective vehicles in complete medium followed by harvesting of the conditioned medium, which was subsequently processed for the isolation of extracellular vesicles.

### 2.2 Extracellular vesicle isolation

Immediately after harvesting the conditioned medium, it was centrifuged at 1,000 rcf for 10 min to remove cells and debris. The supernatant was then filtered through a 0.22 µM tube top filter (Cat# 43,032, Corning) to eliminate apoptotic bodies (~1.0 μm) and reduce the number of microvesicles (>200 nm). The filtrate was transferred into ultracentrifugation tubes using a 10 ml syringe and 18G needle. Initially, 40 ml tubes were used (Cat# 342,414, Beckman) that required heat sealing, but these were later replaced with 34 ml tubes (Cat# 361,625, Beckman) that had convenient caps for sealing. Because a wide range of ultracentrifugation g forces and times were found in the literature, in the majority of experiments, we used an average of these studies, that is, 200,000x g for 2 hr at 4°C employing an Optima™ L‐90K Ultracentrifuge and 70Ti rotor (Beckman). After ultracentrifugation, pellets were identified and the supernatant was gently aspirated and discarded. EV pellets were dissolved in 200 µl of 0.22 μm filtered 1X PBS (Thermo Fisher Scientific), and stored in the −80˚C freezer until further processing. This procedure leads to the enrichment of small‐sized EVs including exosomes (Thery et al., 2018).

### 2.3 Extracellular vesicle quantitation

**Figure 1:**
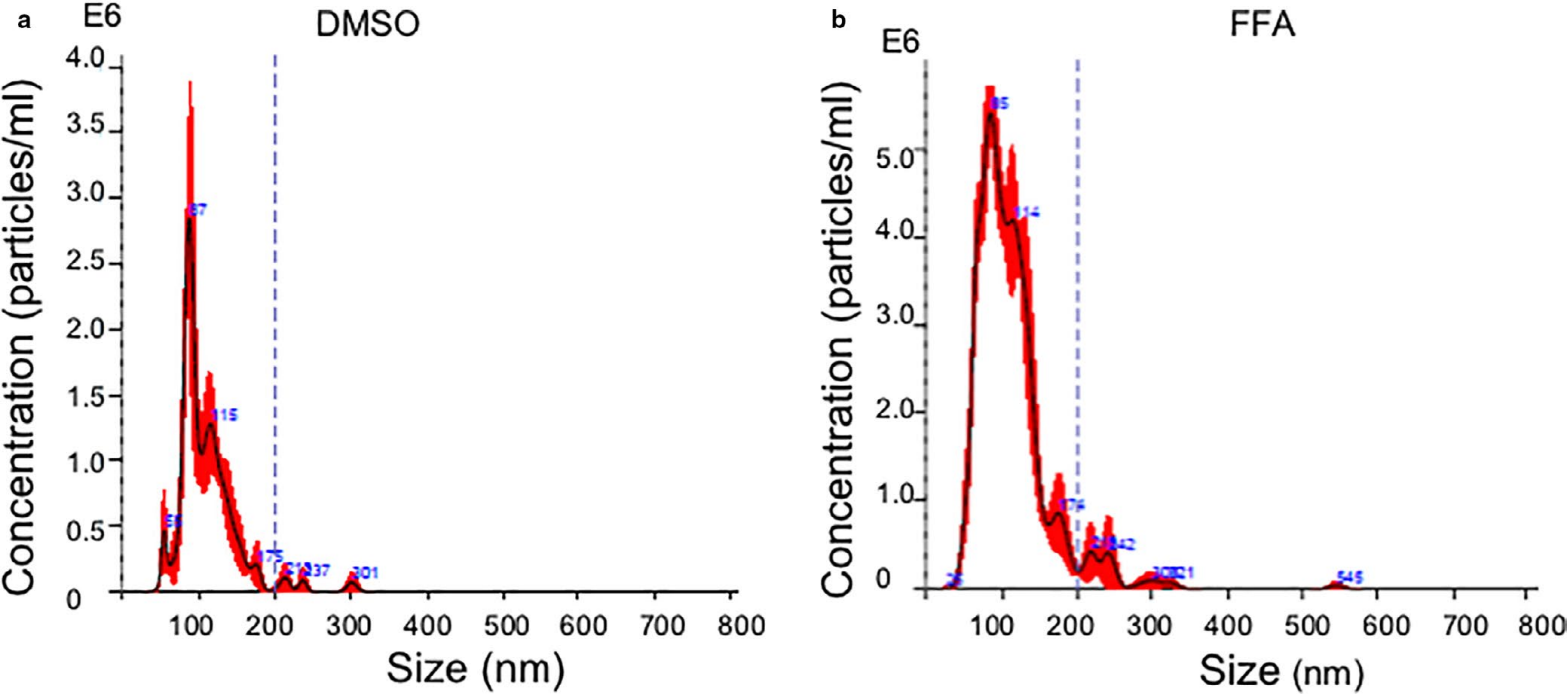
Representative Nanoparticle Tracking Analysis of extracellular vesicles. Representative samples show size (nm) and concentration (particles/ml) of EVs isolated from the conditioned medium of JAR cells that had been treated with (a) dilute DMSO vehicle or (b) 30 μM flufenamic acid (FFA), a TAS2R14 bitter taste receptor agonist, for 24 hr

The isolated EVs were diluted 1/1000 in filtered 1X PBS for quantification in a NS300 NanoSight (Malvern Panalytical) by nanoparticle tracking analysis. Each sample was thoroughly vortexed and drawn up into a 1 ml syringe. About 0.2 ml was then preloaded into the NanoSight through the syringe port. The NanoSight camera focus was adjusted to sharply visualize EVs, and the camera light intensity level was adjusted to appropriate brightness (typically level 14–16). Otherwise, standard measurement settings were selected, and three replicates of each sample were analyzed for a duration of 60 s each. The NanoSight script (protocol) was then run, advancing 0.1 ml of sample per replicate as prompted. The detection threshold was set to 4, in order to allow the NanoSight to quantify the concentration and diameter of EVs during each 60 s recording. Because of the variation in the literature on the range of EV diameters examined (as described in the Introduction), we calibrated the NanoSight so that it would measure EV concentration in the <200 nm range. In 12 experiments, we also measured EV concentration in the <100 nm range for comparison. After quantification was completed, the data from the three replicates were averaged by selecting a multigraph mode. Total concentration was calculated by taking an integral of the area under the concentration curve within the diameter range parameters. Final concentrations were adjusted to account for the initial 1/1000 dilution. The entire analysis and data collection sequence as described were applied to each EV preparation. Figure 1 depicts a representative graph generated by the NanoSight depicting EV diameter on the x‐axis and EV concentration on the y‐axis for flufenamic acid (FFA) and its vehicle control, DMSO (Table 1). Because EV pellets were dissolved in 200 µl of 0.22 μm filtered 1X PBS (vide supra), Figure S1 depicts a nanoparticle tracking analysis of the filtered 1X PBS. The average particle concentration was 2.27 × 10^6^ ± 3.73 × 10^5^ particles/ml.

### 2.4 Extracellular vesicles: validation studies

Four validation approaches were taken to ensure that our preparations were enriched for small EVs.

#### 2.4.1 Syntenin Western Blot

EV preparations isolated from vehicle, FFA, angiotensin II, xanomeline, and sCCK‐treated cells (Table 1) were lysed in RIPA buffer (Thermo Fisher Scientific) containing Halt™ protease inhibitor (1X final concentration; Thermo Fisher Scientific), sonicated, and then loaded into Western blot gels (4%–20% Tris–Glycine gels; Thermo Fisher Scientific). Samples were loaded by volume (5 µl of EV preparation). After the proteins were transferred to a nitrocellulose membrane (Cat# 88,018, Thermo Fisher Scientific), they were incubated with Syntenin antibody overnight at 4°C (2 µl antibody in 10 ml of 5% BSA‐TBS solution for a 1:5,000 dilution or final concentration of 0.2 µg/ml; Cat# ab53522, Abcam). The membrane was then washed and next incubated with goat anti‐rabbit IgG secondary antibody for 1 hr at room temperature (1:3,000 dilution with TBS; cat #170‐6515, Bio‐Rad). The membrane was subsequently washed in 1X Tris‐Buffered saline (TBS; Bio‐Rad), incubated with ECL Select Western Blotting Detection Reagent for 5 min (cat # 2,235, GE Healthcare), and developed using a Bio‐Rad Imager (Image Lab software). The blots were finally incubated with Ponceau S stain (cat # P3504‐10G, Sigma‐Aldrich), in order to reveal total protein in each lane.

#### 2.4.2 Sucrose gradient purification

250 µl of EV preparation from FFA‐treated cells were combined with 250 µl of TNEV‐triton buffer consisting of triton X‐100 (1%; Sigma), 10 mM Tris–HCl, pH 7.5, 150 mM NaCl, 5 mM EDTA, 2 mM sodium vanadate, and Halt protease and phosphatase inhibitor cocktail. Following a 30 min incubation on ice, the contents were mixed with an equal volume of 80% sucrose in TNE (without triton and vanadate) and transferred to a 13 × 23‐mm Beckman centrifuge tube. 35% sucrose in TNE (1,800 µl) was applied to the top of the mixture followed by 5% sucrose (500 μl) in TNE. The sucrose gradient was then ultracentrifuged using an SW 55Ti rotor at 34,000 RPM (110,000 rcf) for 20 hr at 4˚C to separate the contents into 18 fractions of 200 µl volume each based on molecular density. Each fraction was probed for Syntenin on Western blot. Fractions containing EVs corresponded to a density range of 1.12–1.19 g/ml (Colombo, Raposo, & Thery, 2014).

#### 2.4.3 PE‐Adam10 fluorescent labeling

200 µl of isolated EVs were combined with 5 µl of PE anti‐human CD156c (ADAM‐10) antibody (1 µg/ml; Cat# 352,704, BioLegend) in a 2 ml tube. After rocking overnight in a cold room, the contents were ultracentrifuged in a SW 55Ti rotor at 55,000 rpm (280,000 rcf) for 60 min at 4˚C using an Optima™ L‐90K Ultracentrifuge (Beckman). After ultracentrifugation, the supernatant was discarded and the ADAM‐10 labeled EV pellet was resuspended in 200 µl PBS of which 100 µl was diluted with 900 µl of ultrapure PBS for a 10% dilution. PE‐fluorophore has an excitation and emission peaks of 565 nm and 578 nm, respectively. Thus, each sample was processed through the NS300 NanoSight in triplicate for 60 s using a 500 nm fluorescent filter.

#### 2.4.4 Transmission Electron Microscopy (TEM)

Twenty microliters of a 1:10 dilution of each EV sample (vehicle or sCCK) was added to an equal volume of 4% paraformaldehyde in 1X PBS (2% PFA final concentration; Thermo Fisher Scientific). For each sample, a 20 μl drop of the EV‐PFA mixture was dispensed on a clean section of Parafilm, and then a Formvar‐carbon coated grid (Ladd Research Industries; Williston, VT) (membrane side down) was floated on top of the drop. After allowing the membranes to absorb for 20 min, each grid was transferred to a 50 μl drop of 1X PBS before being transferred to a 50 μl drop of 1% glutaraldehyde (Ladd Research Industries; Williston, VT) in 1X PBS and incubated for 5 min. Each grid was subject to a series of eight washes by being transferred and incubated in 50 μl drop of distilled water for 2 min intervals. Then, each grid was incubated in a 50 μl drop of filtered 4% aqueous uranyl acetate (Ladd Research Industries) and 0.15 M oxalic acid (Sigma‐Aldrich) solution pH 7 for 5 min. Each grid was next incubated in a 50 μl drop solution of one part filtered 4% aqueous uranyl acetate and nine parts methyl cellulose (Sigma‐Aldrich) on a section of Parafilm placed in a glass petri dish on a cold plate for 10 min. Finally, the grids were removed with a stainless steel loop and excess fluid was blotted from around the edges with Whatman no. 1 filter paper. After air‐drying the grids for 5–10 min, while still on the loop, the grids were viewed using a Hitachi H‐7600 transmission electron microscope (Hitachi High Technologies America, Inc, Clarksburg, MD) equipped with AMT imaging software (Advanced Microscopy Techniques Corporation).

### 2.5 Cell protein

After harvesting of the conditioned medium, adherent cells were lysed in RIPA buffer containing Halt™ protease inhibitor and vanadate, scraped from the bottom of the flask, and sonicated. Protein concentration was measured by the Pierce BCA Protein Assay Kit (Thermo Fisher Scientific).

### 2.6 Statistical analysis

Results are presented as mean ± *SEM*. Statistics were performed using Graph Pad Prism 7 or 8. Paired samples were compared by the two‐tailed paired *t* test, ratio *t* test (expressed as the geometric mean of ratio ± *SEM* of log ratio), or both. Multiple groups were contrasted by ANOVA. A *p*‐value of < 0.05 was taken to be significant.

## 3 RESULTS

### 3.1 Extracellular vesicles: validation studies

#### 3.1.1 Syntenin Western Blot

**Figure 2:**
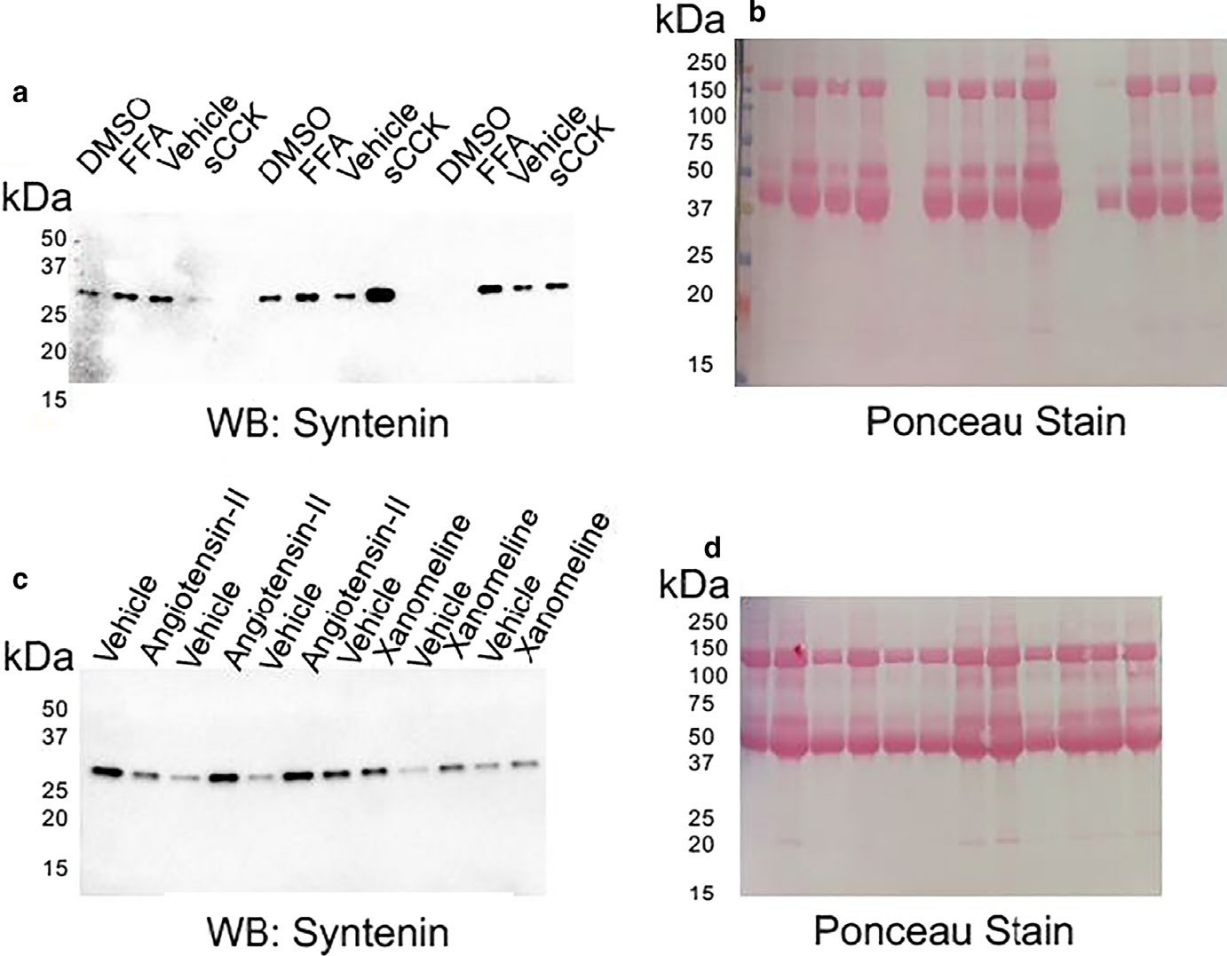
Syntenin western blot of whole extracellular vesicle lysates. (a) and (c) Syntenin western blot; (b) and (d) Ponceau staining. EVs were isolated from the conditioned medium of JAR cells treated for 24 hr with 30 μM flufenamic acid (FFA), 0.1 μM sulfated cholescytokinin (sCCK), 0.1 μM angiotensin II (AII), 3.0 nM Xanomeline (Xan) or vehicle control. See Table 1 for further details of the GPCR agonists and vehicle controls

Syntenin is a prominent protein in small EVs (exosomes) of 33 kDa molecular weight (Kowal et al., 2016). Five μl volume of EVs isolated from cells treated with the various GPCR agonists and their respective vehicles were probed for syntenin on Western blot. With the exception of one vehicle/sCCK pair, there was more immunoreactive syntenin (denser band on Western blot) in EVs isolated from the conditioned medium of JAR cells after the stimulation of the CCKB receptor with sCCK or TAS2R14 receptor with FFA compared to their respective vehicles (Figure 2a). In fact, Ponceau staining was increased for each GPCR agonist versus vehicle treatment (Figure 2b). Because equal volumes of EVs isolated from the conditioned medium of GPCR and vehicle‐treated cells were lysed and loaded into the gel, the denser immunoreactive syntenin and Ponceau bands suggested greater number of EVs after GPCR stimulation consistent with the Nanoparticle Tracking Analysis by NanoSight (as described below).

A Western blot loaded with lysates of EVs isolated from the conditioned medium of angiotensin II and xanomeline‐treated JAR cells were also probed for syntenin (Figure 2c; Ponceau staining: Figure 2d). Syntenin bands were generally denser with xanomeline and angiotensin II treatments compared to their respective vehicles, again consistent with the NanoSight data (as described below). Inexplicably, there was one vehicle/angiotensin II and vehicle/xanomeline pair each, in which the syntenin, but not Ponceau bands were denser in the vehicle lane.

We cannot exclude some loss due to protein degradation even though protease inhibitors were included or some loss of EVs due to the isolation procedure, both of which might have introduced variability in Figure 2. However, we did have sufficient volume for several samples remaining to measure protein concentration by BCA (*n* = 13). We observed a modest but significant relationship between protein and exosome concentration (measured by NanoSight): R^2^ = 0.58, *p* = .0016.

#### 3.1.2 Sucrose gradient purification

**Figure 3:**
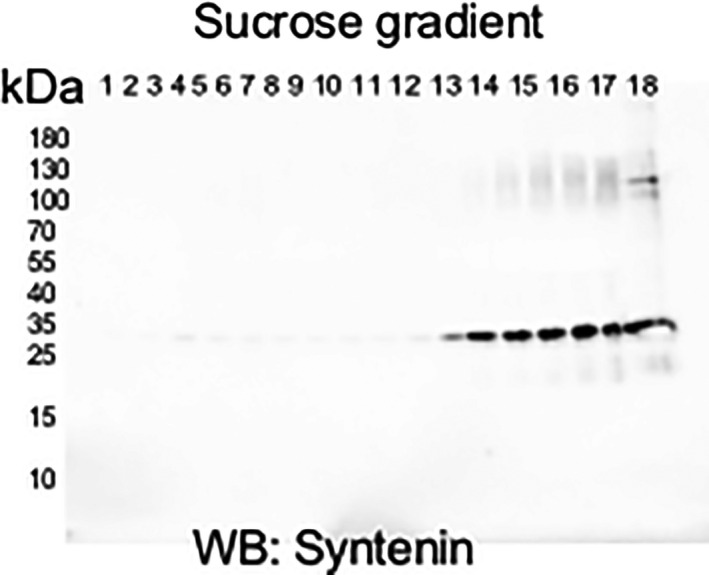
Syntenin western blot of sucrose gradient fractions containing extracellular vesicles. Cultured JAR cells were treated with 30 μM flufenamic acid (FFA; TAS2R14 bitter taste receptor agonist) for 24 hr followed by harvesting of the conditioned medium, which was subsequently processed for isolation of EVs. EVs were further purified by sucrose density gradient ultracentrifugation and then probed for syntenin on western blot

We employed a sucrose gradient to further validate our EV isolation and enrichment methodology. After sucrose gradient ultracentrifugation, EVs are in the last six fractions corresponding to molecular densities of 1.12–1.19 g/ml (Colombo et al., 2014). Syntenin (Kowal et al., 2016) confirmed the presence of exosomes in bands 13–18 by western blot (Figure 3).

#### 3.1.3 PE‐Adam10 fluorescent labeling

**Figure 4:**
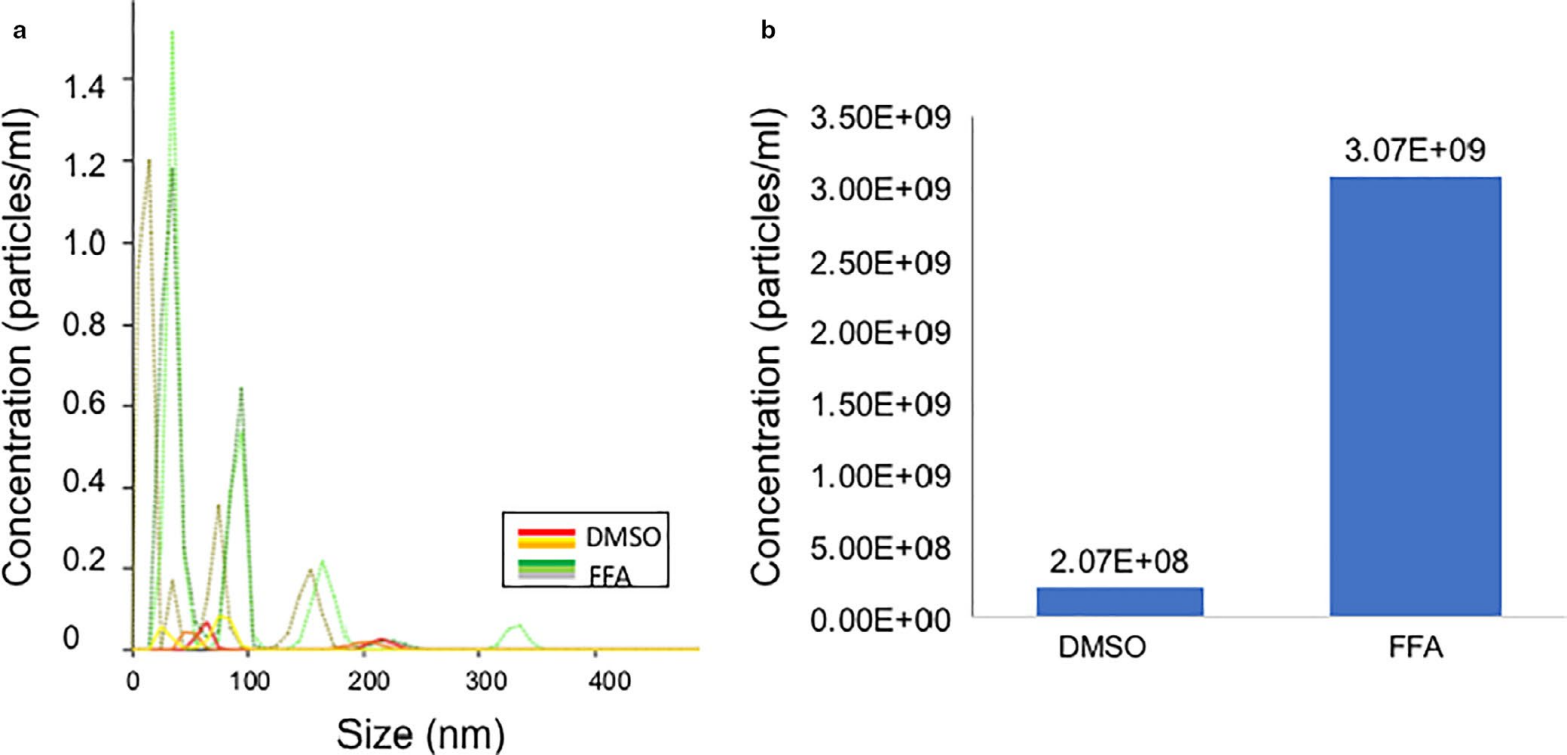
Extracellular vesicle labeling with PE‐ADAM10 antibody. EVs were isolated from conditioned medium harvested from JAR cells after 24 hr treatment with dilute DMSO vehicle or 30 μM flufenamic acid (FFA; TAS2R14 bitter taste receptor agonist). EVs were labelled with fluorescently‐tagged PE‐ADAM10 antibody and quantitated by NanoSight. (a) Depicts three technical replicates each of one biological sample for dilute DMSO (red, yellow, and orange lines) and flufenamic acid (FFA; dark green, light green, and grey lines) treated cells. (b) Average concentration for EVs of < 200 nm

PE‐Adam10 fluorescent labeling was another approach we used to validate the EV isolation technique. PE‐ADAM 10 antibody binds to ADAM 10 found on the phospholipid bilayer of small EVs (Kowal et al., 2016), and of plasma membrane‐derived extracellular vesicles (microvesicles) (Wang & Lu, 2017). Using NanoSight, treatment of JAR cells with FFA and vehicle for 24 hr yielded 3.07 × 10^9^ and 2.07 × 10^8^, respectively, of fluorescently tagged EVs/ml in the conditioned medium (Figure 4). Although the concentration of EVs was a log order magnitude or two lower than measured without PE‐ADAM 10 labeling, this apparent discrepancy maybe explained by inefficiency of PE‐ADAM 10 labeling of EVs, loss of EVs after the second ultracentrifugation step, and the presence of aggregated proteins of similar size in the unlabeled preparations (the latter though not evident on TEM as described next).

#### 3.1.4 Transmission Electron Microscopy

**Figure 5:**
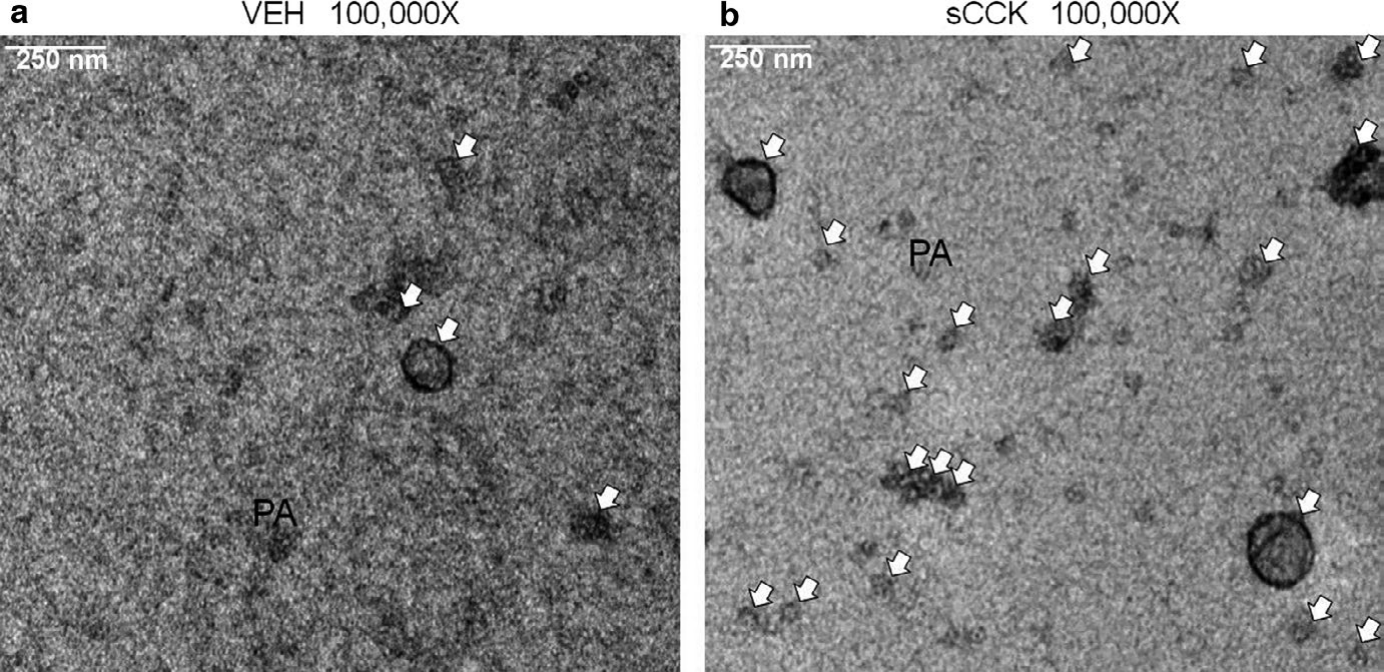
Transmission electron microscopy of isolated extracellular vesicles. TEM was conducted on EVs isolated from the conditioned medium of JAR cells after 24 hr treatment with (a) vehicle for sCCK or (b) 0.1 μM sCCK. White arrows indicate EVs. “PA” denotes protein aggregates

EVs isolated from JAR cells treated with sCCK or vehicle for 24 hr were processed for TEM at 100,000x magnification (Figure 5). TEM differentiated EVs from aggregated proteins by the presence and absence of a phospholipid bilayer, respectively. EVs were qualitatively more abundant after 24 hr of treatment with sCCK in comparison to the vehicle. This final confirmation study further supported a preparation enriched for small EVs (<200 nm).

### 3.2 Multiple GPCRs stimulate EV release

**Figure 6:**
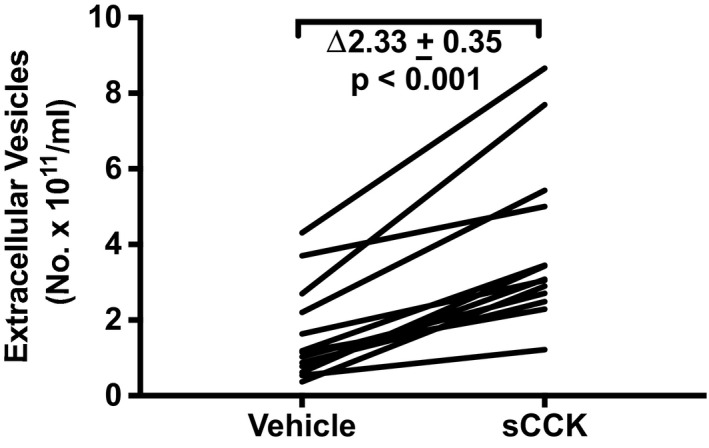
Sulfated cholecystokinin (sCCK: 0.1 μM) stimulated extracellular vesicle release from trophoblast‐derived JAR choriocarcinoma cells. Cultured cells were treated with sCCK for 24 hr followed by harvesting of conditioned medium, which was subsequently processed for isolation of EVs (Methods). *N* = 13 sample replicates in 13 experiments

**Figure 7:**
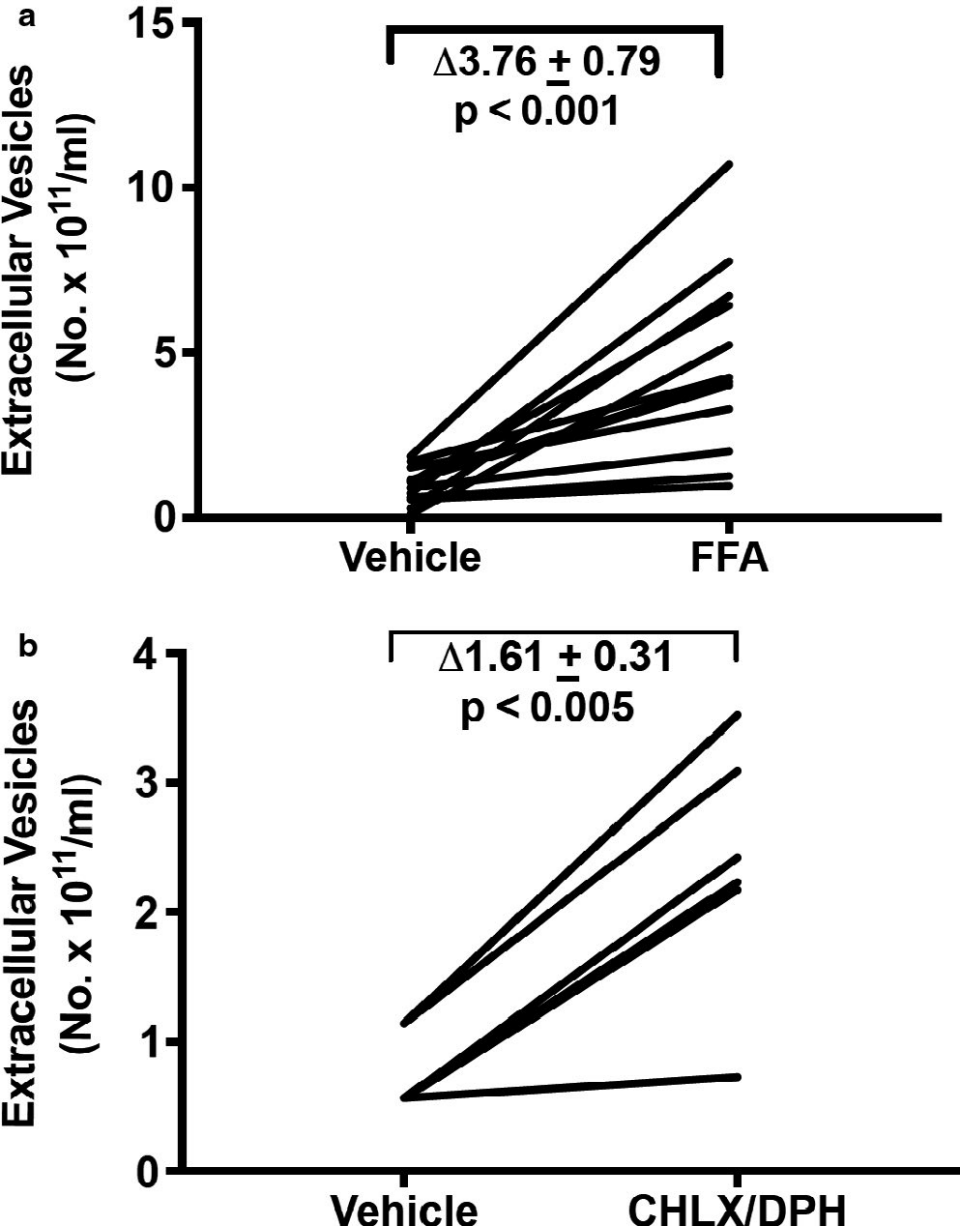
(a) Flufenamic acid (FFA: 30 μM), and (b) Chlorhexidine (CHLX: 10 μM) and Diphenhydramine (DPH: 30 μM) stimulated extracellular vesicle release from trophoblast‐derived JAR choriocarcinoma cells. Cultured cells were treated with FFA, CHLX or DPH for 24 hr followed by harvesting of conditioned medium, which was subsequently processed for isolation of EVs (Methods). *N* = 11 FFA sample replicates in 11 experiments, and 3 CHLX and DPH sample replicates each in 2 experiments

Sulfated cholecystokinin (sCCK) is an agonist of the gastrin/cholecystokinin type B receptor (Taher et al., 2019). When JAR choriocarcinoma cells were incubated with 10^–7^ M sCCK for 24 hr, EV concentration increased by ∆ 2.33 ± 0.35 × 10^11^ particles/ml, when compared to vehicle (*p* = .001; Figure 6). The fold‐increase was 2.79 ± 0.06 (geometric mean of ratio ± *SEM* of log ratio; *p* < .001). Flufenamic acid (FFA), chlorhexidine (CHLX), and diphenhydramine (DPH) are agonists of the human bitter taste receptor, TAS2R14 (Taher et al., 2019). After incubating JAR cells for 24 hr with 30 µM FFA, EV concentration significantly increased by ∆3.76 ± 0.79 × 10^11^ particles/ml compared to vehicle treatment (*p* < .001; Figure 7a) representing a fold‐increase of 4.91 ± 0.13 (*p* < .001). Taken together, 10 µM chlorhexidine and 30 µM diphenhydramine increased EV concentration on average by ∆1.61 ± 0.31 x 10^11^ particles/ml relative to vehicle (*p* < .005; Figure 7b)—a 3.00 ± 0.08 fold‐change in EVs compared to vehicle treatment (*p* < .005).

**Figure 8:**
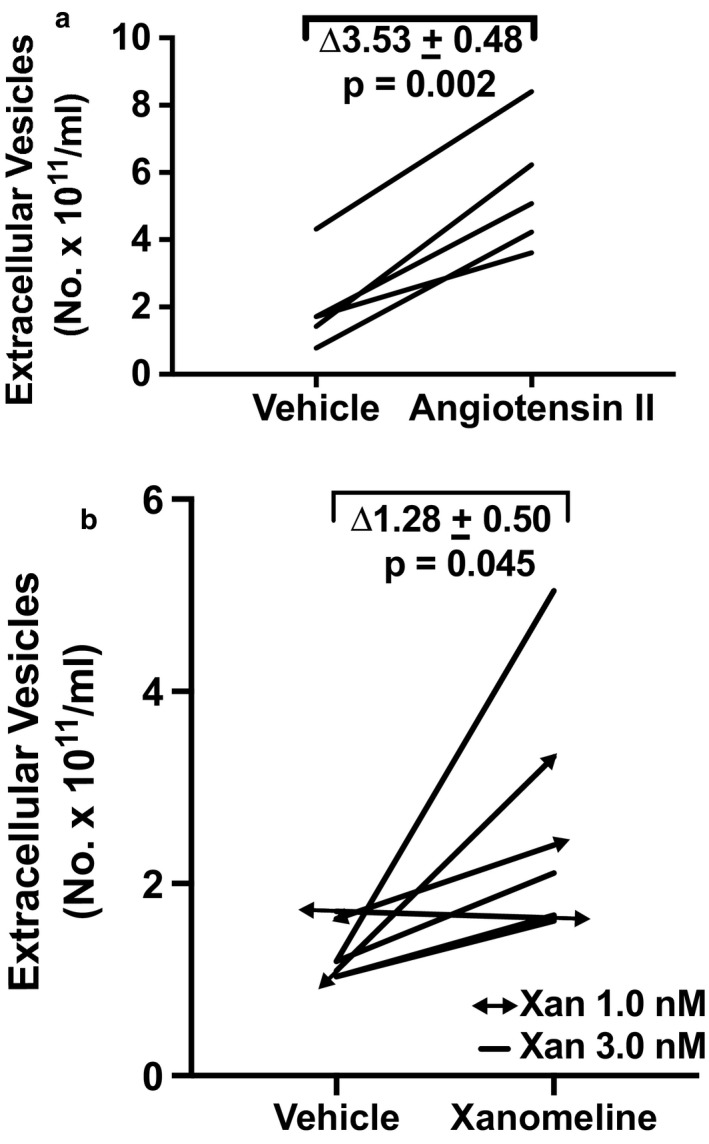
(a) Angiotensin II (AII; 0.1 μM) and (B) Xanomeline (Xan: 1.0 and 3.0 nM) stimulated extracellular vesicle release from trophoblast‐derived JAR choriocarcinoma cells. Cultured cells were treated with Ang II or Xan for 24 hr followed by harvesting of conditioned medium, which was subsequently processed for isolation of EVs (Methods). *N* = 5 Ang II sample replicates in four experiments, and *N* = 7 Xan sample replicates in five experiments

**Figure 9:**
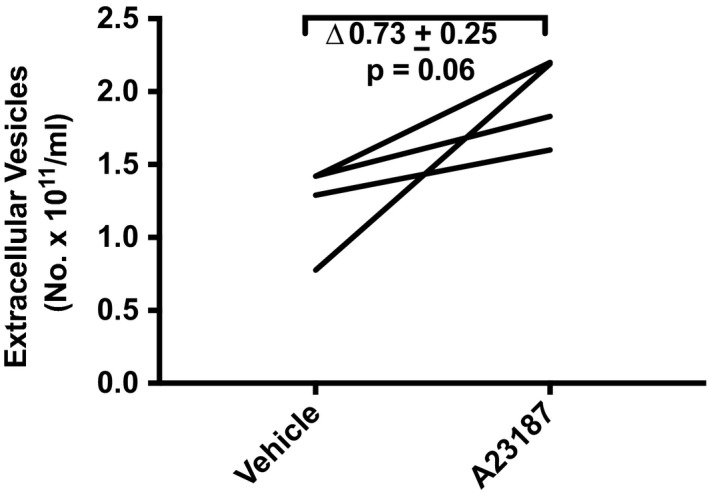
A23187 (3.0 μM) modestly stimulated extracellular vesicle release from trophoblast‐derived JAR choriocarcinoma cells. Cultured cells were treated with A23187, a calcium ionophore, for 24 hr followed by harvesting of conditioned medium, which was subsequently processed for isolation of EVs (Methods). *N* = 4 sample replicates in three experiments

Angiotensin II stimulates angiotensin receptors including the angiotensin II receptor types 1 and 2, as well as the MAS1 receptor after conversion to angiotensin II 1–7. In JAR cells, 10^–7^ M angiotensin II increased EV concentration by ∆3.53 ± 0.48 × 10^11^ particles/ml relative to vehicle treatment (*p* < .005; Figure 8a) representing a fold‐change of 3.11 ± 0.09 (*p* < .005). Xanomeline activates the cholinergic muscarinic receptor subtypes 1/3 [16, 23]. 1.0 and 3.0 nM xanomeline augmented EV concentration by ∆1.28 ± 0.50 × 10^11^ particles/ml compared to vehicle treatment in JAR cells (*p* = .045; Figure 8b). The fold‐increase was 1.87 ± 0.08 (*p* = .015). Finally, EV release was modestly, but not significantly enhanced in JAR cells by 3.0 µM A23187—a calcium ionophore (Figure 9). EV concentration was augmented by ∆0.73 ± 0.25 × 10^11^ particles/ml relative to vehicle (*p* = .06) representing a 1.63 ± 0.08 fold‐change over vehicle (*p* = .08).

### 3.3 Time course of extracellular vesicle release and diameter

**Figure 10:**
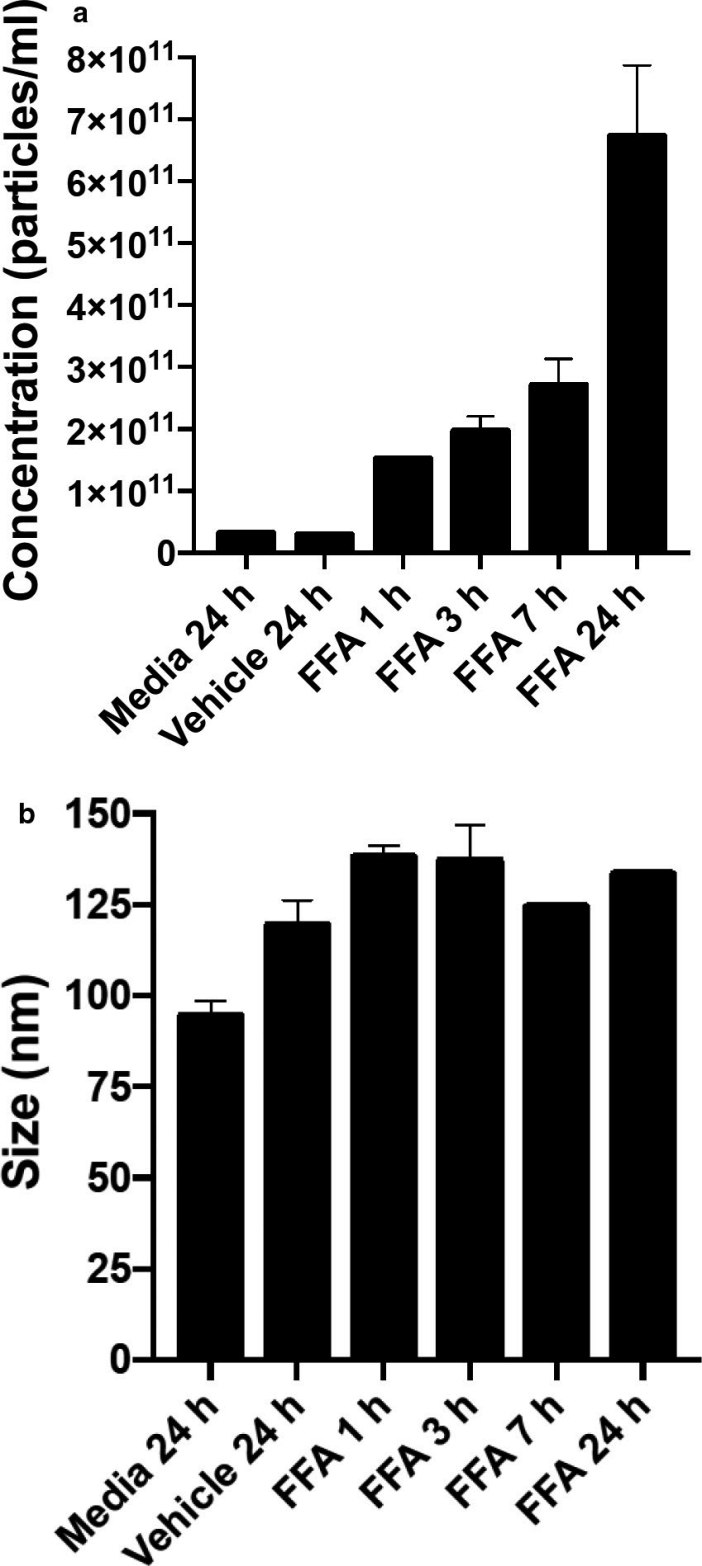
Time course of extracellular vesicle release. (a) EV concentration. (b) EV diameter. EVs were isolated from the conditioned medium that was harvested over a 24 hr time period during flufenamic acid (FFA; 30 μM) treatment. Vehicle treatment was for 24 hr, only. Also shown is EV concentration in complete media alone (without cells) that remained in the cell culture incubator for 24 hr. In some bars, the *SEM* is too small to be visualized. *N* = 1 sample per time point ± *SEM* of triplicate NanoSight determinations.

A time course of EV release was conducted for DMSO‐ and FFA‐treated JAR cells. Conditioned medium was collected at 1, 3, 7, and 24 hr post‐treatment for FFA and at 24 hr only after vehicle treatment. EV concentration progressively rose from 1 to 24 hr (Figure 10a). Because the rise was linear over 24 hr (data not shown), the greatest increase in EV release occurred between 7 and 24 hr. The EV mean diameter at each time point was similar to the dilute DMSO vehicle (Figure 10b).

### 3.4 Effect of the vehicle controls on EV release and diameter

There was no significant difference in the EV concentration (*p* = .13 by ANOVA; Fig. S2a) or mean diameter (*p* = .77 by ANOVA; Fig. S2b) of conditioned medium from JAR cells among the three different vehicles employed following 24 hr of incubation, that is, dilute DMSO, vehicle for sCCK, or cell culture medium, alone (Table 1). Therefore, in some experiments, only one vehicle control was included rather than two or all three.

### 3.5 Effect of GPCR agonist treatments on EV diameter

**Figure 11:**
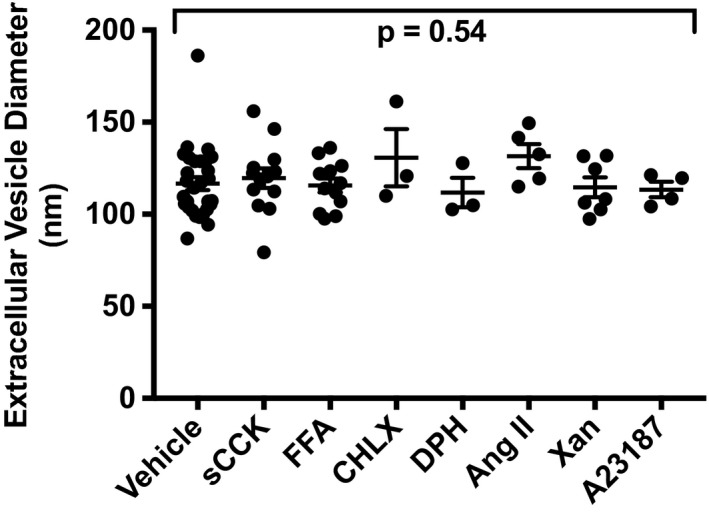
Effect of cell culture medium with or without added vehicles, or containing receptor agonists on extracellular vesicle diameter in the conditioned medium of trophoblast‐derived JAR choriocarcinoma cells. Cultured cells were treated with cell culture medium with or without added vehicles, or agonists for 24 hr followed by harvesting of conditioned medium, which was subsequently processed for isolation of EVs (Methods). Each data point represents one sample replicate

We also analyzed the mean EV diameter for the different treatment groups. Because EV diameters were comparable among the three different vehicles (Fig. S2b), we combined them in this analysis as a comparator group for the receptor agonists. There were no significant differences among the combined vehicles and various agonist treatments in JAR cells (*p* = .54; Figure 11). On average, EV diameter was ~120 nm falling within the anticipated size range of <200 nm for small EVs or exosome‐sized events (Introduction).

### 3.6 sCCK and FFA stimulate EV release from HTR‐8/SVneo cells

**Figure 12:**
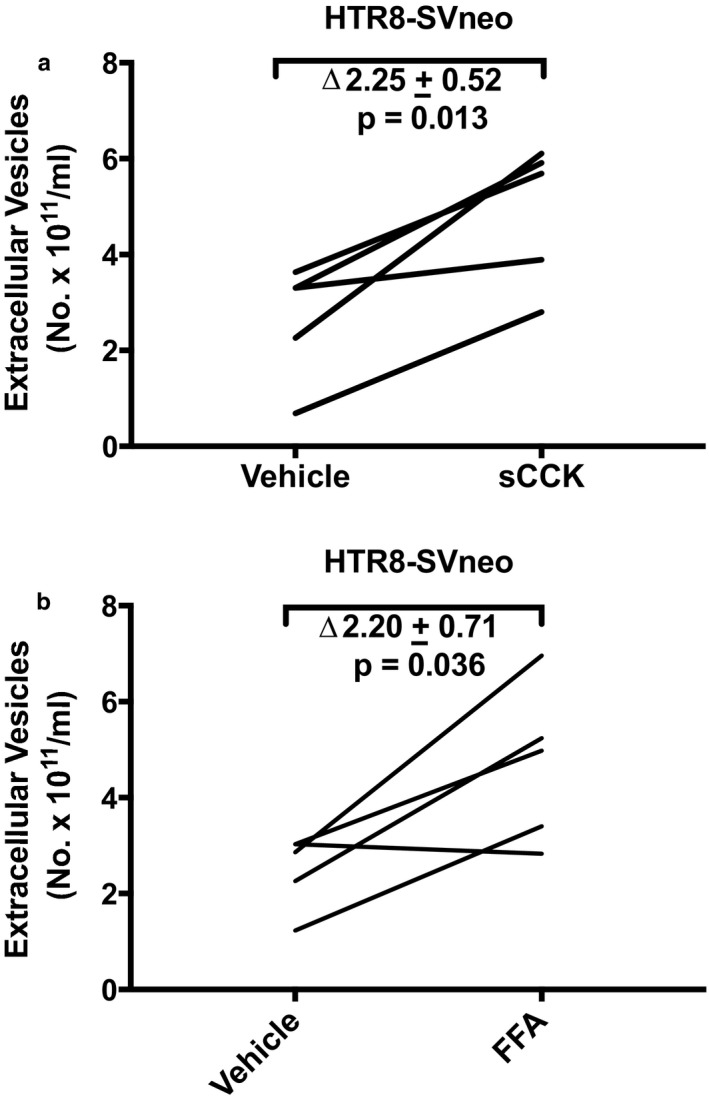
(a) Sulfated cholecystokinin (sCCK: 0.1 μM) and (b) Flufenamic acid (FFA: 30 μM) stimulated extracellular vesicle release from HTR‐8/SVneo trophoblast cell line. Cultured cells were treated with sCCK or FFA for 24 hr followed by harvesting of conditioned medium, which was subsequently processed for isolation of EVs (Methods). *N* = 5 sCCK sample replicates in four experiments, and *N* = 5 FFA sample replicates in four experiments

We also investigated another trophoblast‐derived cell line, HTR‐8/SVneo. When HTR‐8/SVneo cells were incubated with 10^–7^ M sCCK for 24 hr, EV concentration increased by ∆ 2.25 ± 0.52 x 10^11^ particles/ml, when compared to vehicle (*p* = .013; Figure 12a). The fold‐increase was 2.06 ± 0.10 (geometric mean of ratio ± *SEM* of log ratio; *p* = .030). After incubating HTR‐8/SVneo cells for 24 hr with 30 µM FFA, EV concentration significantly increased by ∆2.20 ± 0.71 x 10^11^ particles/ml compared to vehicle treatment (*p* = .036; Figure 12b) representing a fold‐increase of 1.89 ± 0.09 (*p* = .032).

### 3.7 Exosomes: <100 nm versus <200 nm diameter ranges

In 12 experiments using sCCK and vehicle, we also examined the EV diameter range of <100 nm. When compared to <200 nm, the absolute concentration of EVs in the conditioned medium was uniformly less for both vehicle and sCCK, as expected—vehicle: 1.03 ± 0.29 versus 1.99 ± 0.40 x 10^11^ particles/ml, *p* < .005; sCCK: 1.67 ± 0.30 versus 4.04 ± 0.58 x 10^11^ particles/ml, *p* < .001. However, the magnitude of fold‐increase in EV concentration in response to sCCK relative to vehicle was similar for the EV diameter ranges of <100 nm and <200 nm: 2.05 ± 0.23 versus 2.46 ± 0.26, *p* = .096.

### 3.8 Cell protein

Careful documentation of cell layers before harvesting the conditioned medium revealed comparable, virtually complete confluency and no differences in the number of floating dead cells, which were few, in all vehicle and agonist treatments. In seven experiments following vehicle, sCCK or FFA treatments, we lysed and scraped cells from the flasks after harvesting the conditioned medium, and measured the protein concentrations: vehicle 40.3 ± 2.7 (*n* = 14 flasks) and sCCK or FFA 40.7 ± 2.3 mg/ml (*n* = 14 flasks, *p* = .78).

## 4 DISCUSSION

The major finding of this work was that the ligand activation of multiple GPCRs—TAS2R14 bitter taste receptor, gastrin/cholecystokinin type B receptor, cholinergic muscarinic receptor 1 & 3, and types‐1 or −2 angiotensin II receptors—each increased release of extracellular vesicles from trophoblast‐derived cells, but did not alter their diameter. It is possible that in our study angiotensin II increased EV release by activating the MAS1 receptor after metabolism to angiotensin 1–7. Importantly, the enrichment of small EVs in our preparations was validated using four different experimental approaches (Figures 1, 2, 3, 4, 5, Figure S1).

There is a paucity of reports documenting EV release by GPCR activation: AGTR1 in HEK 293T cells, histamine receptors in HeLa cells, and muscarinic receptor type 1 receptor in Jurkat cells (Alonso et al., 2005; Pironti et al., 2015; Verweij et al., 2018). Of note, Pironti et al. further demonstrated that AGTR1 activation increased EV release from cardiomyocytes in vivo (Pironti et al., 2015). Our findings expand the cell‐types in which angiotensin receptor(s) and CHRM1/3 activation lead to EV release to include JAR choriocarcinoma cells—a human trophoblast‐derived cell line (Figure 8).

While the earlier investigations showed EV release by the activation of a single GPCR in the cells‐types described above, our research uniquely addressed the question of whether the activation of multiple GPCRs might release EVs from one cell‐type. Thus, in addition to angiotensin receptor(s) and CHRM1/3 activation, which lead to increased EV release from JAR cells (Figure 8), we further observed that sulfated CCK, a CCKBR agonist (Figure 6), and different agonists of the bitter taste receptor, TAS2R14, also augmented EV release (Figure 7). Comparable results for CCKBR and TAS2R14 were noted in another trophoblast‐derived cell line, HTR‐8/SVneo (Figure 12).

Intracellular calcium was reported to be involved in EV release by many (Chalmin et al., 2010; Faure et al., 2006; Pironti et al., 2015; Savina, Furlan, Vidal, & Colombo, 2003), but not all investigators (Verweij et al., 2018). In our hands, however, 3.0 μM A23187, which we previously showed elicited a 3.5‐fold increase of intracellular calcium on par with the GPCR agonists (Taher et al., 2019), only modestly augmented EV release in JAR cells (Figure 9), the delta‐ and fold‐increase being (considerably) less than the GPCR agonists. In addition, the concentration of DPH that we used to stimulate EV release through TAS2R14 (30 μM; Figure 7b) was 10‐fold or more below the threshold concentration required for raising intercellular calcium in JAR cells (~0.5 mM) (Taher et al., 2019). In the same vein, we observed that sCCK increased EV release in HTR‐8/SVneo immortalized first trimester trophoblast (0.1 μM; Figure 12a)—but sCCK did not increase intracellular calcium in these cells even at a concentration as high as 1.0 μM (Taher et al., 2019). Finally, the 24‐hr time‐course study of EV release by FFA in JAR cells revealed that the greatest rise occurred after 7 hr, indicating perhaps that de novo EV biogenesis, in addition to the secretion of previously formed EVs, may both be important pathways for EV release (Figure 10a). Collectively, these results suggested that other signaling pathways besides, or in addition to, calcium may have contributed to EV release. Indeed, calcium‐independent PKC signaling was implicated in histamine release of EVs from HeLa cells (Verweij et al., 2018), and protein kinase D was identified to be involved in EV release from Jurkat cells by the cholinergic agonist, carbachol (Mazzeo, Calvo, Alonso, Merida, & Izquierdo, 2016). In future studies, the cellular mechanisms of EV release by the ligand activation of GPCRs including the specific G proteins involved and downstream intracellular signaling pathways in trophoblast need to be clarified.

A potential drawback of this study relates to the limitations in our approaches to isolate, enumerate, and investigate EVs of various sizes. In particular, although our preparation was enriched for small EVs, we were not able to clearly discriminate between exomeres, exosomes, and even microvesicles. As such, the various treatments including the vehicle controls and the four different GPCR agonists may have differentially stimulated the release of these three classes of EVs. In order to investigate the biomarkers and biology of specific classes of EVs, we will need to adopt new isolation methods. In the future, this step will be necessary to pursue our next aim, which is to identify the cargos of specific EV types especially exosomes in response to different GPCR agonists.

## 5 PERSPECTIVES

In a previous investigation, we identified expression of the bitter taste receptor TAS2R14, cholecystokinin, gastrin, and their receptor, CCKBR in the human placenta and trophoblast (Taher et al., 2019). In this study, we identified one potential physiological consequence of activating these GPCRs in trophoblast, that is, stimulation of EV release. In addition, we investigated two other GPCRs, which expression has been previously shown in trophoblast cells, CHRM1, AGTR1, and AGTR2 (Pavia et al., 1997; Tower, Lui, Charlesworth, Smith, Aplin, & Jones, 2010). Activation of these GPCRs also increased EV release. Hence, ligand activation of multiple cell receptors expressed in trophoblast enhanced EV release, and albeit purely speculative, perhaps ligand activation of multiple cell receptors in other cell‐types would increase EV release as well.

With as many as 800 GPCRs in the human genome, regulation of EV release by GPCR activation has potential implications for health and disease (Kroeze et al., 2003). The results of the present work make it tempting to speculate that each GPCR (or perhaps cohort of functionally related GPCRs) may augment biogenesis and release of EVs that contain a unique subset of cargo in a cell‐specific fashion, in order to coordinate specific physiological responses among neighboring and distant cells. Conceivably, activation of a specific GPCR that augments the biogenesis and release of EVs with unique cargo could constitute an early disease biomarker (“provocative” liquid biopsy), or provide a therapeutic option by orchestrating neighboring and distant cells to combat disease progression and promote health.

## DISCLOSURES

No conflicts of interest, financial or otherwise, are declared by the authors.

## AUTHOR CONTRIBUTIONS

KPC: study conception and design; AA: study design; CM, EB, KT, KPC: cell culture experiments, EV isolation, and NanoSight measurements; CM, KT, AA: validation studies; KPC, CM: drafted the manuscript; all authors revised and edited the manuscript.

## Supporting information



Fig S1‐S2
