# Supplementary material for: Activation of multiple receptors stimulates extracellular vesicle release from trophoblast cells
Source: Physiol Rep. 2020 Oct 20;8(20):e14592. doi: 10.14814/phy2.14592 (PMC7575225; doi:10.14814/phy2.14592)
Supplement: Supplementary file 1 — Fig S1‐S2 [file PHY2-8-e14592-s001.docx]

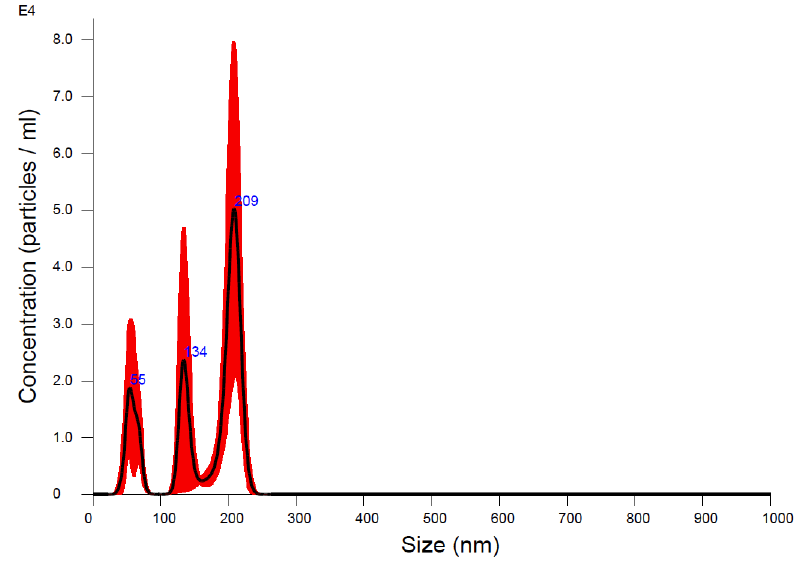


**Supplementary Figure 1. Nanoparticle Tracking Analysis of 0.22 μm Filtered 1XPBS.**

Particles/ml X 10^4^ + SEM of triplicate measurements are displayed as a function of particle diameter (or size in nm). see Methods for further details.

**Supplementary Figure 2. Effect of cell culture complete medium alone, cell culture complete medium with dilute DMSO vehicle or vehicle for sCCK on extracellular vesicle (A) concentration and (B) diameter in conditioned medium of trophoblast-derived JAR choriocarcinoma cells.** Cultured cells were incubated with the various vehicle controls for 24 h followed by harvesting of conditioned medium, which was subsequently processed for isolation of EVs (see Methods).
